# Fulminant myocarditis mimicking catastrophic surgical failure after mitral valve repair

**DOI:** 10.1093/jscr/rjag1025

**Published:** 2026-11-10

**Authors:** Igor Tomczyk, Wojciech Karolak, Aleksandra Ciarka, Aleksandra Stańska, Radosław Targoński, Andrzej Klapkowski, Maciej Brzeziński

**Affiliations:** Department of Cardiac Surgery, Medical University of Gdańsk, Smoluchowskiego 17, Gdańsk 80-214, Poland; Department of Cardiac Surgery, Medical University of Gdańsk, Smoluchowskiego 17, Gdańsk 80-214, Poland; Department of Pathomorphology, Medical University of Gdańsk, Smoluchowskiego 17, Gdańsk 80-214, Poland; Division of Quality of Life Research, Medical University of Gdańsk, Tuwima 15, Gdańsk 80-210, Poland; Department of Cardiology, Medical University of Gdańsk, Smoluchowskiego 17, Gdańsk 80-214, Poland; Department of Cardiac Surgery, Medical University of Gdańsk, Smoluchowskiego 17, Gdańsk 80-214, Poland; Department of Cardiac Surgery, Medical University of Gdańsk, Smoluchowskiego 17, Gdańsk 80-214, Poland

**Keywords:** cardiogenic shock, ECMO, Impella CP, mitral valve repair, tricuspid valve repair

## Abstract

We report a rare case of a 43-year-old woman who developed severe acute left ventricular dysfunction after minimally invasive endoscopic mitral and tricuspid valve repair. Coronary angiography excluded an ischemic complication, while endomyocardial biopsy showed interstitial edema and sparse inflammatory infiltrates without definitive evidence of active myocarditis. Systemic glucocorticoid therapy was initiated after the biopsy result. Temporary mechanical circulatory support with venoarterial extracorporeal membrane oxygenation and Impella CP enabled myocardial recovery and a favorable clinical outcome.

## Introduction

Acute, severe left ventricular dysfunction after cardiac surgery most often results from myocardial ischemia or iatrogenic causes, such as inadequate myocardial protection, air or debris embolization, graft dysfunction, or injury to the circumflex artery during mitral valve surgery. Less common causes involve a different pathophysiology, including myocarditis, which can mimic perioperative complications and pose a significant diagnostic challenge. Fulminant myocarditis (FM) is characterized by a rapid onset of heart failure, often leading to cardiogenic shock; however, with appropriate treatment, it remains a potentially reversible condition [1].

## Case report

A 43-year-old woman with a 10-year history of mitral valve regurgitation was admitted to our Department for mitral valve repair. She also had moderate tricuspid regurgitation and persistent atrial fibrillation; preoperative echocardiography showed reduced left ventricular systolic function (LVEF 45%).

The procedure was performed endoscopically using 3D vision, with cardiopulmonary bypass established via femoral cannulation. Crystalloid cardioplegia with Bretschneider histidine–tryptophan–ketoglutarate (HTK) solution was administered. Mitral and tricuspid valve repairs were performed using annuloplasty rings, and the left atrial appendage was closed with the AtriClip system. Immediately after reperfusion, transesophageal echocardiography (TEE) revealed severe left ventricular dysfunction with near-global akinesis, while right ventricular function remained preserved.

Suspecting iatrogenic injury to the coronary arteries, both rings were removed. A mitral ring (Physio II, size 40) was then re-implanted, and the tricuspid valve was repaired by bicuspidization with a single suture.

Despite these measures, severe left ventricular systolic dysfunction persisted. Venoarterial extracorporeal membrane oxygenation (VA-ECMO) was initiated, and urgent coronary angiography revealed no significant coronary pathology. An Impella CP pump was subsequently implanted to unload the left ventricle. Cardiac biomarkers remained only mildly elevated, arguing against inadequate myocardial protection as the underlying cause, and myocardial stunning was therefore suspected. On postoperative day 6, because cardiac contractility had not improved, an endomyocardial biopsy was performed. At the same time, the Impella pump was explanted in accordance with the manufacturer’s recommendation.

Histopathology showed interstitial edema and sparse inflammatory infiltrates containing eosinophils and neutrophils, without cardiomyocyte damage or definitive evidence of active myocarditis (Fig. 1). After the biopsy result became available, systemic glucocorticoid therapy was initiated. Clinical and echocardiographic improvement was observed shortly thereafter.

**Figure 1:**
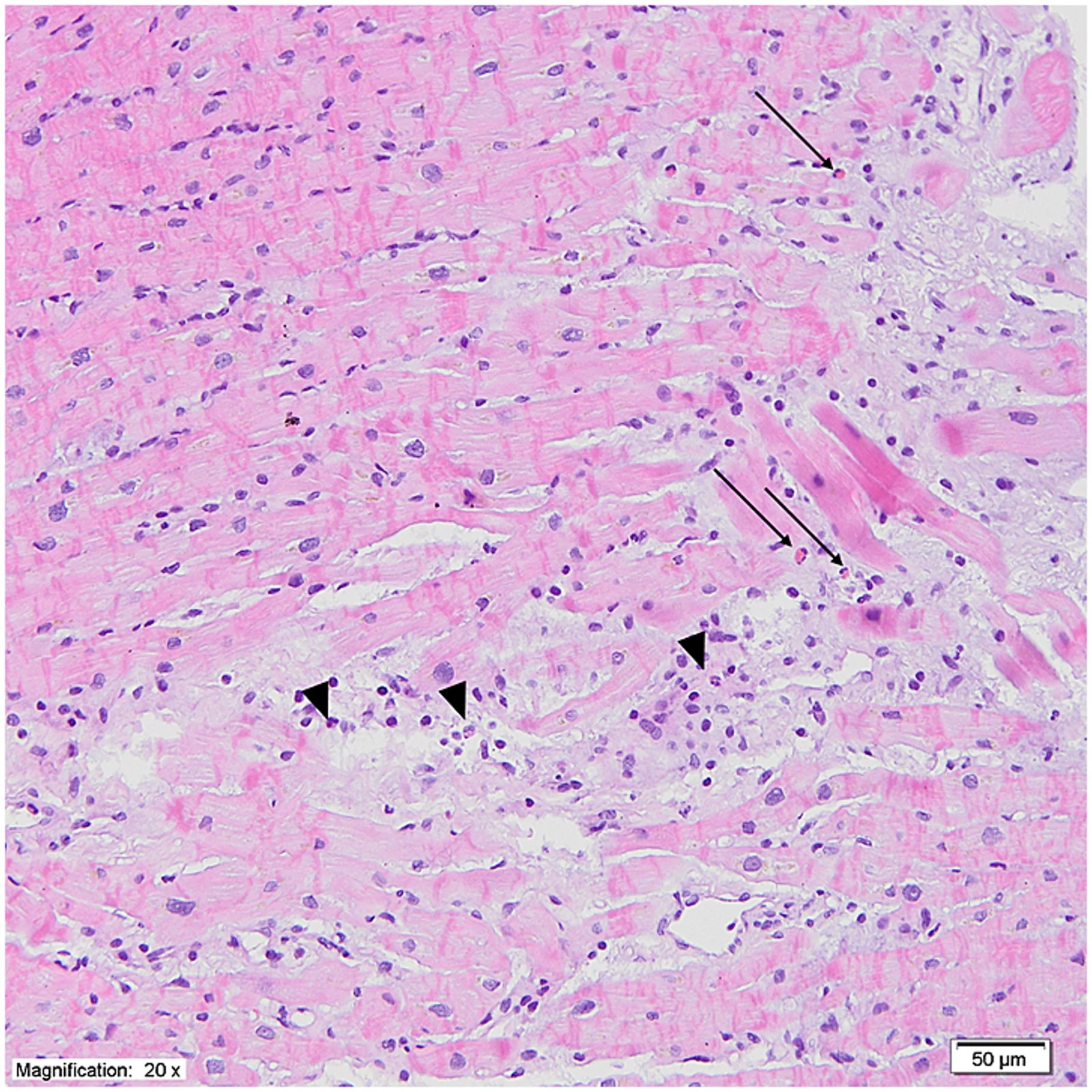
Myocardial tissue with interstitial edema and scattered inflammatory cells, including eosinophils (arrows) and neutrophils (arrowheads), without cardiomyocyte damage. Hematoxylin and eosin staining; original magnification ×20.

On postoperative day 7, a gradual recovery of myocardial contractility was observed; on day 9, ECMO was explanted at an LVEF of ~30%.

The patient was discharged home on postoperative day 12 with an LVEF of 45%, a satisfactory mitral valve repair, and only mild residual tricuspid regurgitation.

## Discussion

This case illustrates the diagnostic difficulty of severe left ventricular failure immediately after cardiac surgery. Endomyocardial biopsy showed interstitial edema with sparse eosinophils and neutrophils but no cardiomyocyte damage and therefore did not fulfil histological criteria for active myocarditis. After surgical and coronary causes had been excluded, the clinical course was considered most consistent with a probable inflammatory myocardial process, including FM [1].

An important alternative is a sterile inflammatory reaction related to cardiopulmonary bypass, ischemia–reperfusion, or cardioplegic arrest. These exposures can activate complement and cytokine pathways, recruit neutrophils, and cause myocardial interstitial edema and inflammatory-cell infiltration [2, 3]. In rare cases, such postoperative changes may overlap with the biopsy appearance of myocarditis despite the absence of an infectious or other external trigger. The immediate onset after reperfusion and the non-specific biopsy findings make this mechanism relevant in our patient. The biopsy alone cannot distinguish a pre-existing or subclinical myocarditis from surgery-related sterile inflammation.

Systemic glucocorticoid therapy was started after the biopsy result, and clinical and echocardiographic improvement followed shortly afterwards. This temporal association is clinically relevant, but it does not establish that glucocorticoids caused the recovery because VA-ECMO and Impella support were being used concurrently. Mechanical circulatory support with the ECMELLA strategy maintained systemic perfusion and unloaded the left ventricle during myocardial recovery. Similar use of combined VA-ECMO and Impella support has been reported in FM complicated by cardiogenic shock [4–7].

The rapid recovery of left ventricular function is compatible with a reversible inflammatory or postoperative myocardial injury. This case therefore supports early exclusion of surgically correctable causes, prompt circulatory support, and cautious interpretation of biopsy findings in the immediate postoperative setting.

FM and a sterile inflammatory response to cardiopulmonary bypass or cardioplegia should be considered when severe ventricular dysfunction follows cardiac surgery and mechanical or ischemic causes have been excluded. Early mechanical circulatory support may provide a bridge to recovery, while biopsy results must be interpreted in their perioperative context.
